# Chronic pain and its correlation with quality of life in hospitalized stable patients with schizophrenia: a cross-sectional survey in Southern Sichuan, China

**DOI:** 10.3389/fpsyt.2026.1772799

**Published:** 2026-07-30

**Authors:** Xing-Ping Tan, Yong-Kui Yang, Xiao-Die Wang, Ren-Tian Guo, Li Liu, Yu-Tong He, Li Liu, Jiang-Lin Wang

**Affiliations:** 1 Department of Pain Management, The Afffliated Hospital, Southwest Medical University, Luzhou, China; 2 Anesthesiology and Critical Care Medicine Key Laboratory of Luzhou, Southwest Medical University, Luzhou, Sichuan, China; 3 Department of Pain Management, People’s Hospital of Jiulongpo District, Chongqing, China; 4 Department of Pain Management, HeJiang People’s Hospital, Luzhou, China; 5 Department of Pain Management, The Fourth Affiliated Hospital, Southwest Medical University, Meishan, China

**Keywords:** chronic pain, pain management, pain perception, quality of life, schizophrenia

## Abstract

The complex paradox of pain perception in schizophrenia often leads to under recognition of chronic pain in this population. To investigate the relationship between chronic pain and quality of life (QoL) in hospitalized stable patients, we conducted a cross-sectional study from November 2024 to April 2025 across three hospitals in Southern Sichuan, China, enrolling 309 patients (mean age 50.3 ± 12.9 years, 84.5% male). For data collection, we employed a self-designed questionnaire and the World Health Organization Quality of Life-BREF (WHOQOL-BREF), the latter being a standardized measure developed by the WHO to assess QoL. Results revealed a chronic pain prevalence of 31.4% (n = 97), predominantly characterized by dull aches in the lower limbs, upper limbs, and head/face. Comparative analysis demonstrated that patients with chronic pain scored significantly lower in physical, psychological, and environmental domains of QoL, along with poorer overall QoL and health satisfaction. This study confirms the high comorbidity of chronic pain among hospitalized schizophrenia patients and its association with comprehensive QoL deterioration. Our findings emphasize the imperative for systematic screening and management of chronic pain in this population as a crucial intervention strategy to improve overall prognosis and QoL.

## 1 Introduction

Schizophrenia is a complex chronic mental disorder that inflicts profound suffering on patients and imposes substantial burdens on families and society (1). Compared to the general population, individuals with schizophrenia endure a heavier burden of life stressors, characterized by limited occupational development, severely impaired social networks, and lower educational attainment, all of which contribute to a significantly reduced QoL. Furthermore, hospitalization rates and illness duration are notably higher in this population (2). While core psychiatric symptoms have been extensively studied, comorbid somatic issues, particularly pain, have long been underestimated (3). This oversight is concerning given that pain is highly prevalent among hospitalized patients across various clinical settings, with reported prevalence rates reaching 71% in Canada, 40-90% in France, and 63% in Germany (4). Given the high prevalence of pain in general hospitalized populations, it is plausible that patients with schizophrenia face a similar, if not greater, burden.

Pain perception in schizophrenia presents a paradoxical phenomenon (5). On one hand, experimental studies using acute pain stimuli (e.g., heat, electrical shock) often report elevated pain thresholds in patients compared to healthy controls, suggesting a state of hypoalgesia (6). On the other hand, other research suggests that the prevalence of chronic pain might be higher in schizophrenia, possibly associated with antipsychotic side effects, comorbid physical conditions (e.g., diabetes, arthritis), or chronic psychological stress. A 2013 cross-sectional study found that patients with schizophrenia were twice as likely to report chronic pain compared to those without the disorder (7).

This discrepancy—between experimental hypoalgesia and potentially higher clinical chronic pain prevalence—creates a critical challenge. According to the International Association for the Study of Pain, chronic pain is defined as persistent or recurrent pain lasting beyond three months (8). Patients with schizophrenia often exhibit cognitive impairments and communication difficulties, leading to underreporting of pain symptoms (9). Additionally, clinicians may engage in “diagnostic overshadowing,” attributing pain complaints to the psychiatric condition itself (e.g., interpreting pain as a somatic hallucination) rather than a genuine comorbid condition (10). Consequently, chronic pain in this population is frequently underestimated, underdiagnosed, and inadequately managed.

This under-identified comorbidity is not benign. Chronic pain can further impair the already compromised QoL of schizophrenia patients through multiple mechanisms (11). Pain directly causes physical discomfort, which may exacerbate negative symptoms such as anhedonia and apathy, reduce motivation for treatment adherence, and increase the risk of depression and suicidal ideation (12).

Despite its potential significance, research on chronic pain in schizophrenia remains relatively scarce, particularly in specific clinical and cultural contexts (13). In China, the prevalence and clinical characteristics of chronic pain among hospitalized patients with schizophrenia are largely unknown, and findings from Western populations may not be directly generalizable due to differences in genetic background, healthcare systems, and cultural attitudes toward pain (14). Furthermore, while the negative impact of chronic pain on QoL is well-established in the general population, studies examining its domain-specific impact—including physical, psychological, social, and environmental domains—in this patient group are lacking. It is also unclear what the current status of pain assessment and treatment is within inpatient psychiatric settings in China.

Therefore, this study aims to address these gaps by conducting a cross-sectional survey among hospitalized patients with schizophrenia in Southern Sichuan, China. Specifically, we sought to estimate the prevalence of chronic pain in this population, describe its clinical characteristics including location, intensity, and current treatment patterns, and investigate the association between chronic pain and QoL.

## 2 Methods

### 2.1 Study design and participants

This multicenter, cross-sectional study was conducted from November 2024 to April 2025 in three psychiatric hospitals in Southern Sichuan, China (covering Luzhou and Zigong cities). It enrolled inpatients who met the International Classification of Diseases (ICD-11) diagnostic criteria for schizophrenia. After rigorous screening, 309 participants completed standardized pain and QoL assessments.

Inclusion criteria were: (1) aged 18–80 years; (2) diagnosed with schizophrenia by a psychiatrist according to the ICD-11; (3) absence of severe physical diseases (e.g., severe cardiac, hepatic, or renal dysfunction); (4) clinically stable condition (defined as no acute psychotic exacerbation in the past week); and (5) voluntary participation with signed informed consent. Exclusion criteria included: (1) comorbid other severe mental disorders (e.g., bipolar disorder, major depressive episode); (2) significant cognitive impairment; (3) pregnancy or lactation. The study was approved by the Medical Ethics Committee of the Affiliated Hospital of Southwest Medical University (Approval No: KY2023016). Written informed consent was obtained from all participants or their legal guardians.

### 2.2 Instruments

The questionnaire comprised three sections. The first section collected basic patient information, including psychiatric diagnosis and socio-demographic data (gender, age, education level, employment status, marital status). The second section assessed pain conditions, including specific clinical manifestations (duration, nature, intensity, accompanying symptoms), relevant medical history, etiology and treatment history of pain, current pain assessment (using a body diagram), and investigation of doctors’ pain management practices. The third section evaluated QoL using the World Health Organization Quality of Life-BREF (WHOQOL-BREF) scale (15).

The WHOQOL-BREF is a standardized instrument developed by the WHO to assess individuals’ QoL. It contains 26 items covering four core domains: physical health, psychological health, social relationships, and environment, plus two independent items evaluating overall QoL and general health. Each item is rated on a 5-point Likert scale. Domain scores are transformed to a 0–100 scale using a standard formula, with higher scores indicating better QoL. The WHOQOL-BREF has demonstrated good reliability and validity and is widely used globally in clinical research, public health, and mental health fields (16).

### 2.3 Procedure

Collaboration was established with psychiatric department medical staff to identify eligible patients meeting the inclusion criteria. The study purpose, questionnaire content, completion methods, and privacy protection measures were explained in detail to patients or their legal guardians. After ensuring full comprehension, written informed consent was obtained.

Prior to questionnaire administration, researchers conducted a detailed review of each patient’s medical records to collect relevant clinical information. This included documentation of comorbid physical conditions (e.g., diabetes, cardiovascular disease), extraction of any pain treatment modalities recorded in the medical history, and other pertinent clinical data. This information was used to supplement and cross-validate data obtained from patient self-reports during the questionnaire session.

Severe cognitive impairment was assessed based on clinical judgment by the attending psychiatrist and the researcher, rather than formal screening tools, to determine whether the patient had significant cognitive deficits that would impair comprehension or participation. Patients with severe cognitive impairment were excluded.

Questionnaires were administered in a quiet, private room to avoid external interference, with each session lasting approximately 30 minutes. Researchers explained the questionnaire items in detail to ensure patient understanding. For patients with limited comprehension, researchers used simple, clear language for explanation, avoiding leading questions. Patients filled out the questionnaires independently. If patients had difficulty reading, researchers provided assistance using a standardized procedure: reading each question and all response options verbatim, recording the patient’s oral response exactly as stated, and reading the recorded answer back to the patient for confirmation. If a response was unclear, contradictory, or appeared unreliable, the researcher asked the patient to clarify or repeat the answer. If significant emotional distress occurred during completion—such as crying, increased anxiety, or verbal expression of unwillingness to continue—the researcher immediately paused the process and notified the attending physician to provide appropriate support and intervention. If the patient’s emotional state stabilized after a brief rest and reassurance, and the patient explicitly expressed willingness to continue, the questionnaire was completed. If the patient remained distressed after rest or explicitly declined to continue, the questionnaire session was terminated, and the patient was excluded from the final analysis.

Researchers entered questionnaire data into an electronic database, with double-checking performed by two independent researchers to ensure accuracy.

### 2.4 Statistical analysis

Data are presented as mean ± standard deviation. Statistical analyses were performed using GraphPad Prism. For continuous variables, non-parametric methods were primarily employed due to data distribution characteristics. Between-group comparisons were conducted using the Mann-Whitney U test, and Spearman’s rank correlation test was used for correlation analyses. Categorical variables are presented as percentages, and group comparisons were performed using the Chi-square test. A *P*-value <0.05 was considered statistically significant.

## 3 Results

A total of 309 patients with schizophrenia were included. The mean age was 50.3 years (SD = 12.9), the majority were male (84.5%), 79.9% were single, 83.8% had an education level of junior high school or below, and 92.9% were unemployed.

**Figure 1:**
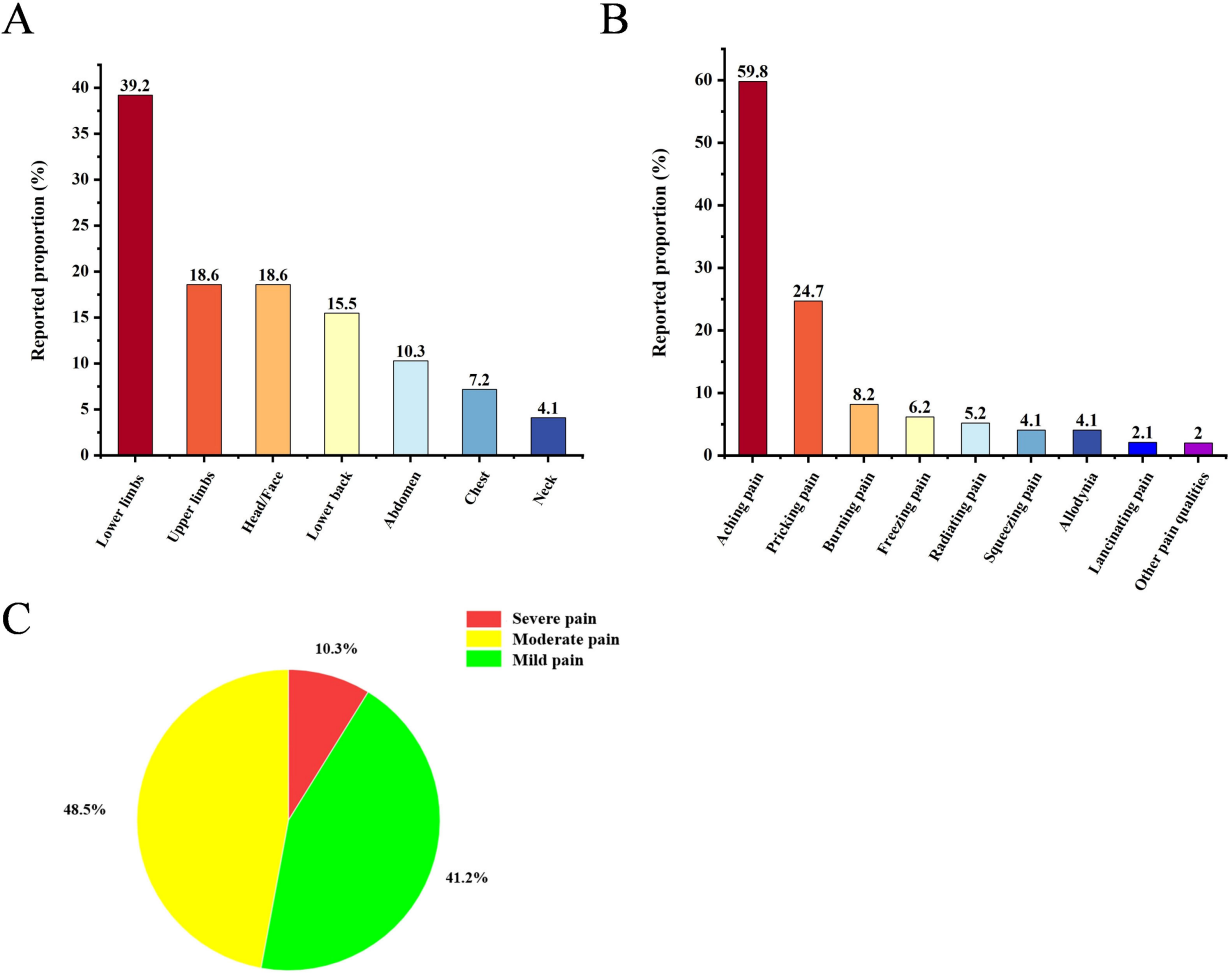
Clinical characteristics of chronic pain in hospitalized patients with schizophrenia (n=97). **(A)** Pain locations. **(B)** Pain types. **(C)** Pain intensity.

Among them, 97 patients reported chronic pain, yielding a prevalence of 31.4%. The most common comorbidities in the chronic pain group were diabetes (17.5%) and cardiovascular disease (17.5%), and 44.3% reported smoking or alcohol use. The primary pain locations were the lower limbs (39.2%), upper limbs (18.6%), and head/face (18.6%)(**Figure 1A**). Aching pain was the most frequent pain type (59.8%), followed by pricking pain (24.7%) and burning pain (8.2%) (**Figure 1B**). The mean pain duration was 39.5 months (SD = 62.1), with moderate pain being most common (48.5%) (**Figure 1C**).

The predominant pain pattern was characterized by intermittent episodes (34.0%), while nearly one-third of patients (28.9%) reported no discernible pain pattern. Pain resolved spontaneously in most patients (37.6%), followed by Western medication (12.8%) and rest (12.8%). Spontaneous exacerbation was the most frequently reported factor for pain aggravation (45.4%), with activity (17.5%), weight-bearing (14.4%), and cold stimulation (14.4%) identified as the primary external triggering factors.

Regarding social support, nearly eighty percent (78.3%) of patients’ families provided limited concern about their pain (41.2% not concerned + 37.1% somewhat concerned). The vast majority (90.7%) of patients with comorbid chronic pain reported that their doctors actively inquired about pain symptoms during medical visits. Over half (54.6%) of the patients with chronic pain received no pain treatment. Among those who received interventions, Western medication (33.0%) and physical therapy (12.4%) were the primary modalities.

**Table 1** compares the baseline demographic characteristics between patients with and without chronic pain. Significant differences were observed in gender (*P* = 0.007) and age distribution (*P* = 0.005). The mean age of the chronic pain group (52.9 ± 13.8 years) was significantly higher than that of the non-chronic pain group (49.1 ± 12.3 years). Additionally, the proportion of female patients was higher in the chronic pain group (23.7%) compared to the non-chronic pain group (11.8%). No other significant differences were observed. These results suggest that gender and age may be associated with the occurrence of chronic pain.

**Table 1:** Sociodemographic characteristics of the study population.

Characteristic	Total (n=309)	Chronic pain		U/*x*^2^	df	*P*
		Yes (n=97)	No (n=212)			
Gender, n (%)				7.205	1	0.007
Male	261 (84.5)	74 (76.3)	187 (88.2)			
Female	48 (15.5)	23 (23.7)	25 (11.8)			
Age (years, *x̄* ± SD)	50.3±12.9	52.9±13.8	49.1±12.3	8244	–	0.005
Age group, n (%)				5.245	1	0.022
18-39	68 (22.0)	15 (15.5)	53 (25.0)			
40-59	177 (57.3)	56 (57.7)	121 (57.7)			
60-80	64 (20.7)	26 (26.8)	38 (17.9)			
Education level, n (%)				0.054	1	0.817
0-9 years	259 (83.8)	82 (84.5)	177 (83.5)			
≥10 years	50 (16.2)	15 (15.5)	35 (16.5)			
Marital status, n (%)				1.123	1	0.289
Single	247 (79.9)	81 (83.5)	166 (78.3)			
Married	62 (20.1)	16 (16.5)	46 (21.7)			
Employment status, n (%)				0.272	1	0.602
Employed	22 (7.1)	8 (8.2)	14 (6.6)			
Unemployed	287 (92.9)	89 (91.8)	198 (93.4)			

QoL domain scores were compared between patients with and without chronic pain (**Table 2**). Except for the social relationship domain (*P* = 0.761), scores in all other QoL domains were significantly lower in the chronic pain group compared to the non-chronic pain group. This result indicates that chronic pain is associated with reduced QoL, particularly evident in the physical health, psychological health, and environmental domains.

**Table 2:** Comparison of quality of life between schizophrenia patients with and without chronic pain.

WHOQOL-BREF domain (*x̄* ± SD)	Chronic pain		U	*P*
	Yes (n=97)	No (n=212)		
Physical	51.4±11.5	61.0±12.5	5653	<0.001
Psychological	48.2±15.4	57.1±14.0	6696	<0.001
Social	41.4±17.0	43.3±19.1	10065	0.761
Environmental	56.8±16.3	63.3±15.8	7984	0.002

Among patients with schizophrenia, those with comorbid chronic pain rated their overall QoL significantly lower and reported lower satisfaction with their health status compared to those without chronic pain (**Table 3**).

**Table 3:** Comparison of general quality of life questions from the WHOQOL-BREF between schizophrenia patients with and without chronic pain.

Question(*x̄* ± SD)	Chronic pain		U	*P*
	Yes (n=97)	No (n=212)		
How would you rate your quality of life?	3.0±0.8	3.2±0.7	8462	0.003
How satisfied are you with your health?	2.9±0.8	3.5±0.8	6443	<0.001

Marital status showed no statistically significant difference in any QoL domain (physical, psychological, social, environmental) among patients with schizophrenia and chronic pain (all *P >*0.05), although numerical trends indicated generally higher scores in the married group (**Table 4**). However, significant differences were observed based on gender: female patients had significantly lower scores than males in the psychological (*P* = 0.022), social (*P* = 0.010), and environmental domains (*P* = 0.006) (**Table 5**). Age showed a significant negative correlation only with the social relationship domain (*P* = 0.016), indicating declining social function with increasing age, but no significant correlation with other domains (*P >*0.05) (**Table 6**). Pain intensity (NRS score) was significantly negatively correlated with the physical health domain (*P* = 0.009) but showed no significant association with other domains (*P >*0.05) (**Table 7**).

**Table 4:** Comparison of quality of life by marital status among schizophrenia patients with chronic pain.

WHOQOL-BREF domain (*x̄* ± SD)	Marital status		U	*P*
	Single (n=81)	Married (n=16)		
Physical	51.2±11.5	52.7±11.7	646.5	0.990
Psychological	47.6±15.1	51.6±17.3	575	0.480
Social	40.7±16.7	44.5±18.2	635	0.899
Environmental	56.3±15.8	59.4±19.0	637	0.917

**Table 5:** Comparison of quality of life by gender among schizophrenia patients with chronic pain.

WHOQOL-BREF domain (*x̄* ± SD)	Gender		U	*P*
	Male (n=74)	Female (n=23)		
Physical	52.0±10.5	49.4±14.2	653	0.091
Psychological	49.8±14.6	43.3±17.2	583.5	0.022
Social	42.9±16.6	36.4±17.6	559.5	0.010
Environmental	58.7±14.6	50.7±20.1	530.5	0.006

**Table 6:** Correlation analysis between quality of life and age in schizophrenia patients with chronic pain.

WHOQOL-BREF domain (*x̄* ± SD)	n	*P*	Correlation coefficient (rs)	95% CI
Physical	97	0.215	-0.127	(-0.324, 0.080)
Psychological	97	0.841	-0.021	(-0.225, 0.185)
Social	97	0.016	-0.245	(-0.428, -0.041)
Environmental	97	0.133	-0.154	(-0.348, 0.053)

**Table 7:** Correlation analysis between quality of life and NRS score in schizophrenia patients with chronic pain.

WHOQOL-BREF domain (*x̄* ± SD)	n	*P*	Correlation coefficient (rs)	95% CI
Physical	97	0.009	-0.263	(-0.444, -0.061)
Psychological	97	0.068	-0.186	(-0.377, 0.020)
Social	97	0.582	0.057	(-0.150, 0.259)
Environmental	97	0.211	-0.128	(-0.325, 0.079)

## 4 Discussion

In this study, the prevalence of chronic pain among hospitalized patients with schizophrenia was 31.4%, predominantly characterized by pain in the lower limbs, upper limbs, and head/face. Compared with patients without chronic pain, those with chronic pain scored significantly lower across multiple domains of QoL (physical, psychological, and environmental), and reported poorer overall QoL and health satisfaction. Furthermore, although 90.7% of physicians actively inquired about pain, over half (54.6%) of patients received no pain treatment, indicating a substantial treatment gap. These findings challenge the traditional view of pervasive hypoalgesia in schizophrenia, highlighting the high comorbidity of chronic pain in this population and its extensive negative impact on QoL (17).

The prevalence of chronic pain among hospitalized patients with schizophrenia in this study was 31.4%, which is close to the 36.6% reported in a Brazilian study, suggesting consistency with certain previous epidemiological findings (18). However, notable variations exist across studies. A Chilean study involving 79 outpatients with schizophrenia reported a chronic pain prevalence of 15.2%, while the prevalence of acute pain was as high as 63% (19). Another cross-sectional study of 1,413 psychiatric patients found that 14% had chronic pain, with the prevalence even lower (8%) among those specifically diagnosed with schizophrenia or other psychoses (18). Conversely, a study conducted in London and Edmonton reported a chronic pain prevalence ranging from 20% to 61% among 78 patients with schizophrenia, comparable to the general population rate of approximately 20% found in that study (18). These discrepancies may be attributed to differences in study populations, sample sizes, pain definitions, and assessment instruments. As an epidemiological investigation conducted specifically in hospitalized patients with schizophrenia in China, this study provides preliminary baseline data on the prevalence and clinical characteristics of chronic pain within this specific inpatient setting. Due to the skewed sample (predominantly male and unemployed), these findings may not generalize to outpatients or female patients.

Our findings stand in stark contrast to the hypoalgesia commonly reported in experimental studies. A substantial body of experimental research using acute pain stimuli such as thermal heat and electrical shock has demonstrated that patients with schizophrenia exhibit higher pain thresholds and greater pain tolerance compared with healthy controls, suggesting a state of hypoalgesia (20). However, the epidemiological data from our study indicate that the prevalence of chronic pain in this population is comparable to or even higher than that in the general population (21). This suggests that acute pain responses observed in laboratory settings cannot be directly extrapolated to the experience of chronic pain in clinical settings. Experimental hypoalgesia may reflect patients’ responses to brief, controlled stimuli, whereas chronic pain involves sustained pathophysiological and psychosocial processes, which may be governed by distinct neural mechanisms. Accordingly, our findings suggest that clinical practice should not underestimate chronic pain in patients with schizophrenia based on experimental evidence of hypoalgesia.

Although self-awareness was not directly measured in this study, this factor may partially explain the clinical phenomenon of pain underestimation in patients with schizophrenia. Previous studies suggest that impaired insight or negative symptoms, such as apathy, may compromise patients’ ability to recognize and articulate their pain experiences (22). Prigatano et al. reported a case of a patient with schizoaffective disorder who denied experiencing pain following acute orthopedic trauma, highlighting the potential impact of impaired self-awareness on pain reporting (23). In the present study, despite patients’ ability to describe the location and nature of their pain to some extent, the severity of pain and the need for treatment may still have been underestimated. This finding suggests that clinicians assessing pain in patients with schizophrenia should consider cognitive function and self-awareness status rather than relying solely on patients’ self-reports.

The risk of comorbid physical conditions is significantly increased in patients with schizophrenia. Prior literature suggests that antipsychotic side effects and prevalent adverse lifestyle factors, such as physical inactivity and poor diet, may contribute to this elevated risk (24). In the present study, cardiovascular disease (17.5%) and diabetes (17.5%) were the most prominent comorbidities among patients with chronic pain. Although patients with schizophrenia may exhibit elevated pain thresholds and increased pain tolerance in experimental settings, previous studies have suggested that this phenomenon may stem from deficits in pain sensitization mechanisms (11). While healthy individuals typically develop pain sensitization following repeated noxious stimuli, patients with schizophrenia—possibly due to dysfunction in the insula-cingulate affective-motivational system—may fail to form normal pain memory reinforcement, leading to apparently elevated thresholds under experimental conditions (25). In contrast, persistent nociceptive input from somatic comorbidities in clinical settings may directly activate the primary somatosensory cortex (S1), potentially contributing to a chronic pain burden comparable to that observed in the general population (26). Taken together, these findings suggest that laboratory pain assessments may not be directly extrapolated to clinical practice, and that the susceptibility of patients with schizophrenia to chronic pain may be underestimated in clinical settings (27).

In the present study, health satisfaction was significantly lower in patients with chronic pain compared to those without chronic pain (2.9 ± 0.8 vs. 3.5 ± 0.8, *P* < 0.001). This finding is consistent with previous studies indicating that chronic pain exerts a substantial negative impact on subjective health perception in patients with schizophrenia (11). The observed reduction in health satisfaction may be attributable to multiple factors, including persistent physical discomfort interfering with daily functioning, the negative emotional impact of pain, and patients’ pessimistic perceptions of their own health status.

Some studies suggest that approximately one-third of patients with schizophrenia exhibit pain insensitivity (11). The long-held notion of pain insensitivity in schizophrenia may not fundamentally stem from diminished pain perception but rather from reduced self-reporting of pain by patients (20). Furthermore, somatic and psychogenic pain are often difficult to distinguish clearly. Regardless of its nature, pain control is crucial as it significantly diminishes QoL (28).

In the present study, although 90.7% of physicians actively inquired about pain, more than half (54.6%) of patients with chronic pain received no pain treatment. This gap between high recognition and low treatment rates may be explained by several clinical factors. First, psychiatrists may lack adequate training and confidence in pain management (29), particularly when treating pain in patients with schizophrenia, due to concerns about potential interactions between analgesics and antipsychotic medications. Second, some patients may hold passive attitudes toward pain, perceiving it as an inherent part of their psychiatric condition and therefore not actively seeking treatment, or may exhibit reduced treatment-seeking behavior due to negative symptoms such as apathy (30). Third, standardized pain management protocols are often lacking in inpatient psychiatric settings, where pain assessment tends to remain at the level of inquiry without being followed by structured treatment pathways or referral mechanisms. Additionally, 41.2% of patients in this study reported that their family members were “not concerned” about their pain, suggesting that inadequate family support may also influence patients’ willingness and opportunity to receive treatment.

Beyond the illness itself, psychopharmacological treatments may also modulate pain responses. Altered sensory thresholds may be partly attributable to psychotropic medications, particularly antipsychotics, whose analgesic properties have been documented (31). Some medications may induce or exacerbate pain. For instance, haloperidol, due to its potent D_2_ receptor blockade, can cause extrapyramidal symptoms, directly leading to muscle rigidity-related pain and headaches (32). Quetiapine’s antagonism of histamine H_1_ receptors might exacerbate dizziness-related pain sensitization (33). Conversely, some antipsychotics possess potential analgesic effects. Among various agents, olanzapine has been shown to possess the most potent analgesic properties (34). Aripiprazole, as a partial agonist at dopamine D_2_ receptors, may indirectly alleviate pain by modulating limbic system function and improving mood symptoms (35).

Taken together, the findings reviewed above—ranging from hypoalgesia and hyperalgesia to the influence of self-reporting and medication effects—highlight the complexity of pain perception in schizophrenia. These contrasting findings provide an important interpretive context for the results of the present study. In our study, 31.4% of patients reported chronic pain and were able to describe its location and quality, which is inconsistent with the traditional view of pain insensitivity and suggests that patients’ ability to perceive and report pain in real-world clinical settings may be more intact than experimental conditions would imply. Taken together, the contradictory literature on pain perception in schizophrenia underscores the complexity of pain in this population. It cautions against dismissing clinical pain based on experimental evidence of hypoalgesia, and against attributing undertreatment solely to deficits in pain perception.

Several limitations of this study should be acknowledged. First, the cross-sectional design allows for the identification of associations but precludes causal inference. Second, significant differences in age and sex were observed between the chronic pain and non-chronic pain groups, yet multivariable regression analyses were not performed to adjust for these potential confounders; consequently, the observed differences in QoL between the two groups may be partially influenced by age and sex rather than attributable solely to chronic pain. Third, pain-related variables were assessed based on patient self-report, which is susceptible to recall bias, particularly in patients with schizophrenia who may have concomitant cognitive impairment that affects the accuracy of their recollections. Fourth, the pain questionnaire used in this study was self-developed and has not been validated against standardized pain instruments, which limits the comparability of our findings with those of studies that employed validated tools. Fifth, the study sample was highly skewed, with 84.5% male, 92.9% unemployed, and all participants being hospitalized patients. This demographic profile does not reflect the broader population of patients with schizophrenia, particularly community-dwelling individuals, those with higher functional status, or female patients. Consequently, our findings may have limited generalizability to outpatient settings, female-dominated populations, or patients with better occupational and social functioning.

Beyond the scope of the present study, several avenues for future research merit consideration.

First, in addition to traditional clinical epidemiological investigations, future research may employ bibliometric methods to map the research landscape, emerging trends, and developmental trajectories of pain and its influencing factors in hospitalized patients. For example, a bibliometric analysis of the top 100 cited articles on ankylosing spondylitis provided valuable insights into the knowledge structure and evolution of that field (36).

Second, with the widespread use of digital technologies, hospitalized patients increasingly seek pain relief information online, a trend with dual implications for the doctor-patient relationship (37). Online resources may enhance patient engagement, but variable information quality can foster mistrust or unrealistic expectations. Previous studies have shown that online materials for chronic low back pain and AI-generated medical texts often have quality and readability issues (38). Clinicians should therefore explore patients’ information sources, help them identify reliable content, and guide them toward evidence-based pain management strategies to build trust and collaboration. Future research could further examine the impact of social media use on pain perception and treatment adherence in schizophrenia.

Despite these limitations, this study demonstrates a high burden of chronic pain among hospitalized stable patients with schizophrenia in Southern Sichuan, China, and its significant negative association with multiple domains of QoL. Notably, although the vast majority of physicians (90.7%) actively inquired about pain, more than half (54.6%) of patients with chronic pain received no treatment, suggesting that the core issue is not pain identification, but rather the lack of effective intervention following identification. Therefore, these findings underscore the importance of integrating a systematic pain management process—rather than merely pain assessment—into routine care for schizophrenia. This should include standardized pain screening, clear treatment pathways, multidisciplinary collaboration, and training for psychiatrists in pain management, aiming to translate pain recognition into effective intervention and thereby improve the overall prognosis and QoL in this vulnerable population.

## Data Availability

The original contributions presented in the study are included in the article/supplementary material. Further inquiries can be directed to the corresponding authors.
